# The optimal cut-off score of the Eating Attitude Test-26 for screening eating disorders in Japan

**DOI:** 10.1007/s40519-024-01669-1

**Published:** 2024-06-08

**Authors:** Nobuhiro Nohara, Maiko Hiraide, Takeshi Horie, Shu Takakura, Tomokazu Hata, Nobuyuki Sudo, Kazuhiro Yoshiuchi

**Affiliations:** 1 Department of Stress Sciences and Psychosomatic Medicine, Graduate School of Medicine, The University of Tokyo, 7-3-1, Hongo, Bunkyo-Ku, Tokyo, 113-8655 Japan; 2 Department of Psychosomatic Medicine, Kyushu University Hospital, 3-1-1 Maidashi, Higashi-Ku, Fukuoka, 812-8582 Japan; 3 Department of Psychosomatic Medicine, Graduate School of Medical Sciences, Kyushu University, 3-1-1 Maidashi, Higashi-ku, Fukuoka, 812-8582 Japan

**Keywords:** EAT-26, Cutoff, Eating disorders, Feeding and eating disorders, Anorexia nervosa, Bulimia nervosa

## Abstract

**Purpose:**

The Eating Attitude Test-26 (EAT-26) is a screening tool for eating disorders (EDs) in clinical and non-clinical samples. The cut-off score was suggested to be varied according to target population. However, no studies have examined the appropriateness of the originally proposed score of 20 for screening DSM-5 eating disorders in Japan. This study aimed to identify an appropriate cut-off score to better differentiate clinical and non-clinical samples in Japan for EDs.

**Methods:**

The participants consisted of 54 patients with anorexia nervosa restricting type, 58 patients with anorexia nervosa binge-eating/purging type, 37 patients with bulimia nervosa diagnosed according to DSM-5 criteria, and 190 healthy controls (HCs). Welch’s *t* test was used to assess differences in age, body mass index (BMI), and total EAT-26 scores between HCs and patients with EDs. Receiver operating characteristic (ROC) analysis was conducted to identify the optimal cut-off score.

**Results:**

The HCs had significantly higher BMI and lower total EAT-26 mean scores than patients with EDs. The area under the ROC curve was 0.925, indicating that EAT-26 had excellent performance in discriminating patients with EDs from HCs. An optimal cut-off score of 17 was identified, with sensitivity and specificity values of 0.866 and 0.868, respectively.

**Conclusions:**

The result supports the suggestions that optimal cut-off score should be different according to target populations. The newly identified cut-off score of 17 would enable the identification of patients with EDs who have been previously classified as non-clinical samples in the EAT-26 test.

*Level of evidence*: III: evidence obtained from case–control analytic study.

## Introduction

The Eating Attitude Test-26 (EAT-26), which was developed as an abbreviated version of the original EAT-40 [1], is one of the most widely used measures of symptoms of eating disorders (EDs) and serves as a screening tool for non-clinical populations [2, 3]. The reliability and validity of both the original and the Japanese version of the EAT-26 have been demonstrated [2, 4–7]. The factor structure of the EAT-26 has been found to be different depending on the target populations [8–17], and the optimal cut-off value was suggested to be varied according to the target populations [4, 8, 12, 18–20].

So far, only one study by Nakai [21] has examined the appropriateness of the cut-off score of 20 suggested by the original EAT-26 [3] for screening disordered eating or possible patients with EDs in Japan, which proposed an optimal cut-off score of 15. However, their clinical ED samples were diagnosed according to DSM-IV criteria, and both clinical and non-clinical samples consisted of those aged between 15 and 36 years. Moreover, despite the proposal of the cut-off score of 15, the original cut-off score of 20 has been widely used in Japan [22–30].

The characteristics of patients with EDs have been found to differ between in Japan and in Western countries. Japanese patients with EDs have been observed to exhibit less perfectionism and less drive for thinness compared to those in Western countries [31–33]. Considering the differences in eating disorder profiles between Japanese and those in other countries, and changes in diagnostic criteria from DSM-III to DSM-5 [8–16], the cut-off value of 20 proposed by the original study [3], or that of 15 (as proposed by Nakai) [21] may not be applicable for screening EDs based on the DSM-5 criteria in Japan.

Therefore, the current study aimed to identify an optimal cut-off score for the EAT-26 that could better differentiate clinical ED samples from non-clinical samples in Japan.

## Methods

This study was conducted as per the principles of the Declaration of Helsinki. The study protocol was approved by the ethics committees of the Graduate School of Medicine at the University of Tokyo and Kyushu University. All the participants gave their consent for participation in this study.

### Participants

In this study, the EAT-26 scores were collected in person as part of the cross-sectional and observational study to investigate the psychometric properties of the fear of food measure (FOFM) in Japanese women in 2018 [28]. In that study, all the patients were recruited from University of Tokyo Hospital and Kyushu University Hospital. To be eligible for the study, participants needed to be16 years old or older, had been diagnosed with anorexia nervosa restricting type (AN-R), anorexia nervosa binge-eating/purging type (AN-BP), or bulimia nervosa (BN) according to DSM-5 criteria and were in treatment by experienced clinicians specialized in EDs. A total of 152 patients with EDs were recruited in that study, however, data for three patients were missing and therefore excluded from the analysis. Then, the patient group consisted of 54 patients with AN-R, 58 patients with AN-BP, and 37 patients with BN. No cases of binge eating disorder (BED) were identified in this sample.

A total of 208 healthy female controls who had never received any psychological or pharmaceutical treatment for any disease were recruited according to the age groups of past outpatients with EDs from the University of Tokyo Hospital (16–19, 20–29, 30–39, and 40–49 years) in a 2:4:3:1 ratio from a pool of approximately 10 million registered individuals residing in internet research company, Macromil, Inc. They were ineligible to the study if they had a BMI of less than 17.5 kg/m^2^ or had amenorrhea without taking oral contraceptives, which were indicative of an individual with EDs, to exclude possible patients with ED [6]. Eighteen individuals with a BMI of less than 17.5 kg/m^2^ were excluded from recruited samples and a total of 190 women were incorporated in the study. There were no missing data for healthy women.

### Measures

Eating attitudes were measured using the Japanese version of the EAT-26 [5]. The original version of the EAT-26 was developed to differentiate people with EDs from those without them, and was validated as a screening instrument in non-clinical settings and as an outcome measure in clinical groups [1, 3]. It is a self-reported questionnaire, comprised of 26 items each rated on six-point Likert scale (0 = “never,” “rarely,” or “sometimes”; 1 = “often”; 2 = “usually”; and 3 = “always”). Possible scores range from 0 to 78, and a cut-off score of 20 was proposed to classify a similar proportion of AN and HCs.

### Statistical analysis

Welch’s *t* test with a significance level of 0.05 was used to assess differences in age, BMI, and total EAT-26 scores between HCs and patients with EDs. Receiver operating characteristic (ROC) analysis was conducted to examine the optimal cut-off values of the EAT-26 for discriminating between HCs and patients with EDs, and the sensitivity and specificity corresponding to the optimal cut-off values were calculated. All analyses were conducted using R version 4.2.0 and R package ROCR version 1.0-11 [34].

## Results

### BMI, age, and EAT-26 total scores of HCs and patients with EDs

Table 1 summarizes the age, BMI, and total EAT-26 scores of the participants. There were no significant differences in age between HCs and patients with EDs; however, the HCs had a significantly higher mean BMI and lower mean EAT-26 total score than patients with EDs.

**Table 1:** Age, BMI, and total EAT-26 scores of HCs and patients with EDs

	HC (*n* = 190)	ED (*n* = 149)	*p* value	Effect size:* d*	95% CI
	Mean (SD)	Mean (SD)			
Age	28.7 (8.26)	30.0 (10.4)	0.20	0.14	[− 0.07, 0.36]
BMI*	21.1 (2.85)	17.4 (5.04)	< 0.001	0.92	[0.69, 1.14]
Total EAT-26 score*	8.48 (7.71)	35.0 (17.0)	< 0.001	2.10	[1.83, 2.36]

ED: Patients with eating disorders, including AN-R, AN-BP, and BN. Effect size: Cohen’s *d*. *CI:* confidence intervals of effect size

*Significant difference between HC and ED groups (*P* < 0.05)

### Optimal cut-off value and performance characteristics

**Fig. 1:**
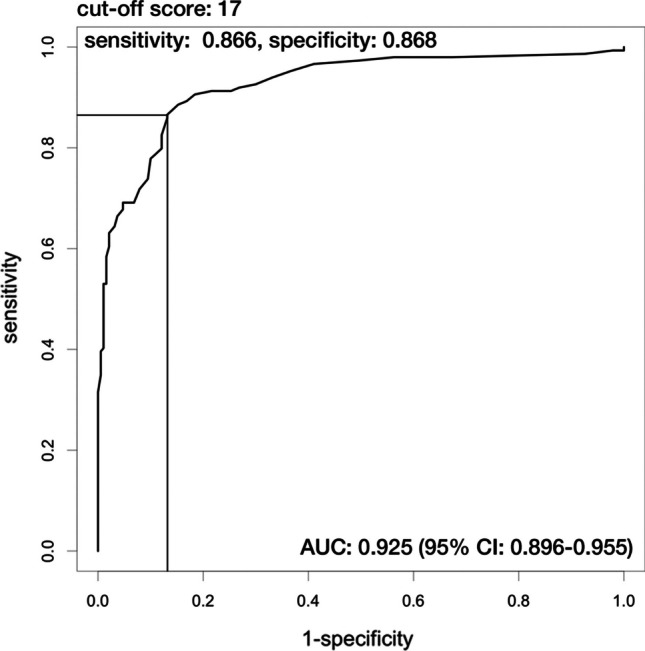
ROC curve for the EAT-26 scores of the participants (the figure was generated by R version 4.2.0. Lettering to the figure was added by Adobe Photoshop Elements 2020)

Figure 1 shows the ROC curve of the EAT-26 in the study population. The area under the curve (AUC) was 0.925 (95% CI 0.896–0.955), indicating that EAT-26 had excellent performance in discriminating patients with EDs from HCs. A cut-off score of 17 was found to best maximize Youden’s index with sensitivity and specificity of 0.866 and 0.868, respectively. The positive predictive value (PPV) was 0.838, the negative predictive value (NPV) was 0.892, the positive likelihood ratio (LR+) was 6.58, and the negative likelihood ration (LR−) was 0.155. A total of 129 clinical and 25 non-clinical samples scored at or above the identified cut-off score.

Cronbach’s alpha values of the EAT-26 were 0.90 (95% CI 0.87–0.92) for the clinical samples and 0.83 (95% CI 0.79–0.86) for the non-clinical samples, indicating that the EAT-26 exhibited satisfactory inner consistency for the target populations.

## Discussion

This study aimed to identify an optimal cut-off value for the EAT-26 for discriminating between HCs and patients with EDs in the general Japanese population. The results indicated that a cut-off value of 17 best discriminated patients with EDs from HCs, and the PPV was 0.838 when using the identified cut-off score. Considering the low prevalence rates of AN and BN among young women in Japan, reported as 0.43% and 2.32%, respectively [35], it is plausible that the EAT-26 with the identified cut-off score is effective in distinguishing between individuals with EDs and non-clinical individuals in Japan. Moreover, the proposed cut-off score of 17 would support the notion that the optimal cut-off score should be set based on specific populations [4, 8, 12, 18–20], while it was challenging to completely dismiss the influence of changes in diagnostic criteria for EDs.

Diagnostic criteria for EDs have been mitigated over time. The original study used a modified version of the Feighner Criteria to diagnose patients with AN [3, 36]. The Feighner Criteria and criteria of DSM-III, DSM-IV, and DSM-IV-TR for AN are more stringent than those defined in DSM-5 with removal of amenorrhea and elimination of the threshold for determining low weight [37, 38]. Therefore, the current study likely included patients with AN who would have not been diagnosed with AN according to the previous diagnostic criteria. Indeed, application of the DSM-5 criteria led to an increase in the number of patients diagnosed with AN, along with a decrease in the proportion of eating disorder not otherwise specified (EDNOS) identified based on DSM-IV [39–41]. Therefore, the optimal cut-off score was lower than that of the original study [3].

The optimal cut-off value identified in the present study was higher than that proposed for the Japanese population in a previous study, despite both studies targeting Japanese population [21]. An interpretation is that in the current study, all participants in the ED group were recruited from two centers: the University of Tokyo Hospital and Kyushu University Hospital. Both hospitals are one of the largest tertiary care teaching hospitals in Japan, and most patients were referred from primary or secondary care hospitals because of the severity of EDs. Consequently, EAT-26 scores in the ED group in the present study were more likely to be higher than those treated in primary or secondary care hospitals, which could cause that the optimal cut-off score was higher than that of the previous study, in which participants in the ED group were treated at both Kyoto University Hospital and the university-affiliated hospitals [21]. Moreover, the previous study did not provide information on the mean and standard deviation of the participants’ age distribution. Therefore, differences in ages between patients with EDs and HCs in their study might be one of the reasons why their optimal cut-off value differed from that of the present study.

The identified optimal cut-off score for the EAT-26 in Japan was lower than that of 23 in Spain [12]. It has been reported that female patients with AN in Japan, as well as in Hong-Kong, exhibited non-fat phobic profiles [33, 42, 43], and especially female patients with AN-R in Japan exhibited less drive for thinness compared to those in Western countries [33]. These characteristics may have contributed to lowering the EAT-26 scores in patients with EDs and the optimal cut-off score. In addition, previous study has revealed that the EAT-26 score significantly increased with increasing BMI [44]. While the BMI distribution of the participants in the Spanish study was not explicitly stated, the average BMI of Spanish women in 2018 was 25.6 [45], which was higher than that of the target population in this study, potentially contributing to lowering the optimal cut-off score.

The identified optimal cut-off score of 10 for the EAT-26 in Oman was lower compared to that in Japan [20]. The traditional aestheticism in Oman, which characterized plumpness as socially desirable and a symbol of fertility [46], might cause lower EAT-26 scores in Oman population. Moreover, inclusion of men in target population might have influenced the lowering of the EAT-26 scores. It has been demonstrated that male individuals are likely to score lower in the EAT-26 compared to females [47].

The identified optimal cut-off score for the EAT-26 in Japan was higher than that of 15 in China mainland [4]. Three possible explanations can be considered for the difference in optimal cut-off values. First, the ED group in their study included patients with BED or other specified feeding or eating disorder (OSFED). The total EAT-26 scores of patients with BED or OSFED were lower than those of patients with AN or BN in their study, potentially influencing the optimal cut-off score. Second, both the mean age and BMI of patients with EDs in their study were lower than those of the participants in the present study, which may contribute to differences in the EAT-26 score distributions between the studies. Third, it is conceivable that cultural differences between Japan and China contributed to the disparity in the optimal cut-off score, despite both studies comprising non-Western participants. As mentioned in the Background, the presentation of disordered eating attitudes and behaviors is sensitive to cultural differences [48–53]. From these considerations, it would be possible to delineate the culturally influenced dispersion of the EAT-26 scores by controlling for demographic variables such as age, gender and BMI of the target population.

In addition, the significance of the EAT-26 as a screening tool should be considered. The criterion of “There should be an accepted treatment for patients with recognized disease” outlined in the WHO’s screening guideline underscores the importance of the availability of effective treatments when making screening decisions [54, 55]. Therefore, with the current lack of definitive curative treatment for ED, the significance of screening for EDs would warrant careful consideration. However, several promising treatments have been proposed in guidelines, such as NICE guideline and APA guidelines [56, 57], enabling the initiation of treatment based on screening results. Moreover, earlier treatment was shown to be associated with more favorable prognosis [58]. Therefore, EAT-26 could be used as a screening tool for EDs.

## Strength and limits

The strength of the study is that an optimal cut-off score for the EAT-26 was identified that could better differentiate clinical ED samples from non-clinical samples in Japan. Considering the paucity of studies on EDs in non-western countries [59], the strength of the study might also lie in mentioning the differences in characteristics of patients with EDs in Japan and other countries through the exploration of the difference in the optimal cut-off value of the EAT-26. However, several limitations should be considered.

First, it is important to note that the HC group did not fully represent the Japanese population. While non-clinical samples in the HC group were randomly collected without restriction on their residential region, the age distribution was intentionally aligned with that of clinical samples, limited to ages 16–49. Consequently, it was not proportional to that of the Japanese female population. However, considering that age of onset for EDs falls mostly between the mid-10s to mid-20s [60–62], the usefulness of the proposed cut-off value would not be compromised by the limitation.

Second, there is possibility that some patients with EDs were misclassified as HCs. In the present study, the HC group exclusively comprised individuals devoid of any history of EDs or other psychiatric disorders. However, given that their past medical history was solely based on self-report, it would be appropriate to consider that individuals in the HC group constituted putative healthy samples rather than healthy samples. Moreover, participants in the HC group were selected based on the condition that their BMI was equal to or above 17.5, and cessation of menstruation without oral contraceptives was not accompanied to exclude possible patients with AN from HC samples. The conditions could not entirely exclude patients with AN or BN from HC group because the BMI of patients with BN was above 18.5, and some patients with AN had a BMI above 17.5 without amenorrhea [63]. However, it is known that those with EDs are likely to avoid self-report surveys to assess their ED pathology or eating-related problems by not agreeing to participate [64]. Therefore**,** the percentage of misclassified participants in the HC group may be small in this study.

Third, the present study did not assess the impact of perceived stress or the negative effects of depression and anxiety on the EAT-26 scores. It is possible that the severity of comorbid depression or bipolar disorders in patients with EDs partially affected their EAT-26 scores and the optimal cut-off values. Previous studies have revealed that total and subscale scores of the EAT-26 were significantly affected by depressed mood and anxiety through disordered-eating [16, 65–70]. Mood and anxiety disorders is common psychiatric comorbidities in patients with EDs, and the prevalence rate of mood spectrum disorders, especially bipolar disorders, is higher among patients with BN compared to those with AN [61, 71–75]. Indeed, the impact of stress or the negative effects of depression and anxiety on the EAT-26 scores is not a limitation specific to this study, but rather, an inherent limitation of the EAT-26 questionnaire itself. Therefore, some previous studies evaluated the Beck Depression Inventory second edition (BDI-II) or state trait anxiety inventory (STAI) concurrently with the EAT-26 to assess the impact of those psychological states on EAT-26 scores [11, 67, 76]. Considering the methodology in those previous studies, the lack of concurrently assessment of these mental state in the study could be pointed out as a limitation, because we may have been able to propose a more appropriate cut-off value with these assessments. To rigorously ascertain the usefulness of the proposed cut-off score, a replication of the study is warranted, incorporating an assessment of the influence of mood states.

### What is already known on this subject?

An optimal cut-off score of 15 for the EAT-26 was identified for screening disordered eating or possible patients with EDs in Japan [21]. However, their clinical ED samples were diagnosed according to DSM-IV criteria, and the age range in the samples was limited between 15 and 36 years.

### What this study adds?

The study identified an optimal cut-off score of 17 for the EAT-26 for screening EDs based on the DSM-5 criteria in Japan.

## Conclusions

A cut-off score of 17 was found to be optimal for the EAT-26 as a screening tool for EDs in the Japanese population, which might be able to reduce the number of untreated patients, because the original cut-off score of 20 was widely used in Japan.

## Data Availability

The data sets analyzed during the current study are available from the corresponding author on reasonable request.
